# Supplementary material for: BPKEM: A biometric-based private key encryption and management framework for blockchain
Source: PLoS One. 2024 Mar 4;19(3):e0286087. doi: 10.1371/journal.pone.0286087 (PMC10911611; doi:10.1371/journal.pone.0286087)
Supplement: Attachment — Submitted filename: Response to Reviewers.docx [file pone.0286087.s001.docx]

**Response to Reviewers**

**Dear Sir/Madam,**

**Thank you very much for all your comments. We made substantial changes in response to your comments, and fell the quality of this paper had a great improvement as a result of updating this paper taking your suggestions. Thank you very much for your valuable comments and please check our updates in response to each comment:**

**Response to academic editor:**

**Point 1:** Please ensure that your manuscript meets PLOS ONE's style requirements, including those for file naming.

**Response 1:** Thanks for your suggestion. We double-checked the format of the manuscript and modified the format of relevant figures and tables to meet PLOS ONE's style requirements.

**Point 2:** Please note that PLOS ONE has specific guidelines on code sharing for submissions in which author­generated code underpins the findings in the manuscript. In these cases, all author-generated code must be made available without restrictions upon publication of the work.

**Response 2:** Thanks for your suggestion. We shared the code about biometric feature extraction and generating stable and distinguishable descriptors on github, and the access link of the code is: https://github.com/liiihan/BPKEM. Since the size of the blockchain part exceeds the shareable size limit, it cannot be shared on GitHub. If you want to get unlimited codes for codes, please contact us by email: xujianlong@stu.edu.cn

**Point 3:** We note that you have provided funding information that is not currently declared in your Funding Statement. However, funding information should not appear in the Acknowledgments section or other areas of your manuscript. We will only publish funding information present in the Funding Statement section of the online submission form. Please remove any funding-related text from the manuscript and let us know how you would like to update your Funding Statement.

**Response 3:** Thanks for your suggestion. We have removed any funding-related text from the manuscript.

**Point 4:** In your Data Availability statement, you have not specified where the minimal data set underlying the results described in your manuscript can be found. PLOS defines a study's minimal data set as the underlying data used to reach the conclusions drawn in the manuscript and any additional data required to replicate the reported study findings in their entirety. All PLOS journals require that the minimal data set be made fully available.

**Response 4:** Thanks for your suggestion. The datasets we use are all public datasets and are completely open. We have added relevant links about the public datasets we used in the revised cover letter.

**Point 5:** We note that Figure 5 includes an image of a patient participant in the study. If you are unable to obtain consent from the subject of the photograph, you will need to remove the figure and any other textual identifying information or case descriptions for this individual.

**Response 5:** Thanks for your suggestion. We have checked the relevant pictures, the pictures used are the pictures in the public data set, and the publisher has authorized.

**Response to reviewer 1:**

**Point 1:** There is no performance analysis in this paper. Both security and efficiency are the necessary indices to assess the value of a work. However, no performance analysis is found in this paper. As a consequence, the superiority of the proposed protocol in performance is not demonstrated.

**Response 1:** Thanks for your suggestion. We have added section 5 to analyze the security and performance of the scheme. We briefly analyze the security of the authentication scheme of the proposed framework and compare it with other schemes, and calculate the storage cost and computation cost of the scheme

The changes are reflected in lines 432 to 500.

**Point 2:** In Related work, the authors don't explain clearly why they use the main technology they have presented. What's more, the authors just simply show the references' main idea and they don't analyze these references and explain the development of this main technology.

**Response 2:** Thanks for your suggestion. We have supplemented some of the analysis of references in the related work, and also added supplements to the paper on the private key authentication phase, and explained the reasons for proposing BPKEM at the end of the section.

The changes are reflected in lines 73 to 152.

**Point 3:** In section 4.3.1, the authors propose a universal and stable distinguishable descriptor extraction method. However, they don't give a specific explanation. And the given schematics can't explain well about the methodology. There are many other similar methods similar to what this paper shows. So, the authors should give a detailed and clear explanation, not a few similar words.

**Response 3:** Thanks for your suggestion. We have supplemented the specific explanation of the stable and distinguishable descriptor extraction method, by extracting feature points to generate feature point coordinates in the two-dimensional image coordinate system, and then obtaining stable and distinguishable descriptors by calculating the two-dimensional control points of the feature points. We have reworked the relevant flow charts to make the description of the differentiable descriptor extraction method clearer and easier to understand

The changes are reflected in lines 268 to 319.

**Point 4:** There are some typos in this paper. For example, in contributions, the sentence “we propose A comprehensive management solution for the blockchain's private key system ...…“ should be "we propose a comprehensive management solution for the blockchain's private key system ..…. “ Please carefully check this paper and correct similar errors.

**Response 4:** Thanks for your suggestion. We double checked the paper and corrected some typos in the paper.

**Point 5:** Table and pictures in this paper are not standard. It is suggested that the author check the format of the full text to improve its readability of the full text.

**Response 5:** Thanks for your suggestion. We have checked the format of the full text, and modified non-standard diagrams to improve the readability of the full text.

**Response to reviewer 2:**

**Point 1:** Section 3 must be strengthened.

**Response 1:** Thanks for your suggestion. We supplemented the concept and principle of the fuzzy extractor in Chapter 3, and added an explanation about LWE.

The changes are reflected in lines 154 to 214.

**Point 2:** section 5 must include more experimental graphical results.

**Response 2:** Thanks for your suggestion. We added some related charts in Section 6, and added two metrics, Average Hamming distance and Decline rate. In Experiment 1, we added a description table of face feature extraction points and the experimental data table of experiment on stable discriminative descriptors based on facial features. Added Supplementary instructions on fingerprint enhancement and the experimental data table of experiment on stable discriminative descriptors based on fingerprint features. In Experiment 3, the equipment, Ethereum creation block parameter table and private chain system diagram are supplemented.

The changes are reflected in lines 532 to 676.

**Point 3:** some of the following interesting works on block chain must be reviewed

1.	"A secure and efficient authentication and data sharing scheme for Internet of Things based on blockchain."

2.	"BBAAS: Blockchain-Based Anonymous Authentication Scheme for Providing Secure Communication in VANETs."

3.	"EAAP: Efficient Anonymous Authentication With Conditional Privacy-Preserving Scheme for Vehicular Ad Hoc Networks."

**Response 3:** Thanks for your suggestion. We reviewed papers 1 and 2 in the related work in Section 2, and compared the corresponding schemes of 1, 2 and 3 in the security performance analysis of Table 1.

The changes are reflected in lines 122 to 136 and Table 1.
